# Supplementary material for: Generalizing clusters of similar species as a signature of coexistence under competition
Source: PLoS Comput Biol. 2019 Jan 22;15(1):e1006688. doi: 10.1371/journal.pcbi.1006688 (PMC6358094; doi:10.1371/journal.pcbi.1006688)
Supplement: S1 Text — (PDF) [file pcbi.1006688.s001.pdf]

Generalizing clusters of similar species as a signature of coexistence under competition

Rafael D'Andrea[1,2*], Maria Riolo[2], Annette M Ostling[2,3]

1 Ecology and Evolutionary Biology, University of Michigan, Ann Arbor, Michigan, USA

2 Plant Biology, University of Illinois, Urbana-Champaign, Illinois, USA

* rdandrea@illinois.edu

S1 Text: Glossary

Cluster: a set of species with similar trait values and relatively high abundance, separated from other such sets by sparsely populated regions in trait space.

Competition-colonization tradeoff: a niche mechanisms commonly invoked for plant coexistence. Species may offset disadvantages in one-on-one contests for recruitment via superior ability to colonize freshly available habitat, or vice-versa [1].

Ecological drift: ecological analogue to genetic drift, whereby changes in the abundance of a species occur via random sampling from a finite population, independent of fitness or competitive standing related to species traits [2].

Limiting similarity: a classical theoretical concept that has taken on multiple meanings over time [3, 4], here interpreted as greater-than-chance trait differences between adjacent species on the niche or trait axis. While competing species were traditionally expected to display limiting similarity, the demonstrated robustness of clusters to demographic stochasticity, their persistence under immigration, and their appearance under disparate niche mechanisms suggest that clusters may be more common than limiting similarity.

Mass effects: phenomenon in source-sink dynamics whereby a locally disfavored population can be sustained via immigration from populations favored elsewhere [5].

Niche, niche strategy, niche differences: a concept in community ecology pertaining to idiosyncrasies in how a species responds to and in turn affects the environment, resources, and/or other species [6]. Traditionally used to describe species differences in environmental preferences or in strategies for procuring resources, avoiding enemies, etc. In modern coexistence theory, "niche differences" are operationally defined as species differences that stabilize competitive coexistence, which requires stronger competition within than between species [7,8]. Stable competitive coexistence occurs when each species can grow from low abundance in the presence of the others.

Niche axis: the axis of interspecific variation pertaining to niche strategies and niche differences. For example if insectivorous bird species differ in their preference for prey of different sizes, preferred prey size would form a niche axis. In contrast, in the competition-colonization tradeoff, the axis runs from competition specialist (high-quality low-number seeds) to colonization specialist (low-quality high-number seeds).

Niche mechanism: any set of circumstances that stabilizes coexistence, typically by ensuring that competition is stronger within species than between them [8]. In a niche mechanism, the nature of species interactions, plus sometimes the presence of tradeoffs, lead to opportunities for species to differ in their interaction with limiting factors [9]. Examples include specialization to different prey and/or different abiotic environments.

Optimal niche strategy: In a closed community, when the current number of species exceeds the number of species that can stably coexist, only those with optimal niche strategies will persist indefinitely. Optimal niche strategies are sufficiently separated from one another on the trait axis to enable stable coexistence. The particular set of optimal niche strategies that emerges from the competitive process can be determined in part by external circumstances such as which

resources are in greater supply, or simply by the starting conditions or order of arrival of species to the community. Mathematically, models of competition among species on niche axes typically have a pattern-forming instability [10], whereby species with optimal strategies become increasingly abundant while all others dwindle towards exclusion.

Stabilization vs. equalization: a dichotomy in modern coexistence theory [8] whereby competitive exclusion can only be mitigated via mechanisms that either stabilize the community (niche mechanisms), or reduce average fitness differences between species (equalizing mechanisms). While the concept is a useful theoretical construct, it can be difficult or impossible to calculate the degree of stabilization and equalization in multispecies communities, or to assign trait differences to one or the other [11] (but see [12] for an update to the theory).

References

1. Levins R, Culver D. Regional Coexistence of Species and Competition between Rare Species. Proceedings of the National Academy of Sciences of the United States of America. 1971;68(6):1246–1248.

2. Hubbell SP. The Unified Neutral Theory of Biodiversity and Biogeography. Princeton, NJ: Princeton University Press; 2001.

3. Abrams P. The Theory of Limiting Similarity. Annual Review of Ecology and Systematics. 1983;14(1):359–376. doi:10.1146/annurev.es.14.110183.002043.

4. D'Andrea R, Ostling AM. Challenges in linking trait patterns to niche differentiation. Oikos. 2016;125(10):1369–1385. doi:10.1111/oik.02979.

5. Shmida A, Mark V Wilson. Biological Determinants of Species Diversity. Journal of Biogeography. 1985;12(1):1–20. doi:10.2307/2845026.

6. Chase JM, Leibold M. Ecological Niches: Linking Classical and Contemporary Approaches. The University of Chicago Press; 2003.

7. Chesson P. A Need for Niches ? Trends in ecology & evolution. 1991;6(1):26–28.

8. Chesson P. Mechanisms of maitenance of species diversity. Annual Review of Ecology and Systematics. 2000;31(2000):343–358.
9. Levin S. Community equilibria and stability, and an extension of the competitive exclusion principle. The American Naturalist. 1970;104(939):413–423. doi:10.2307/2678832.
10. Pigolotti S, López C, Hernández-García E. Species Clustering in Competitive Lotka-Volterra Models. Physical Review Letters. 2007;98(25):1–4. doi:10.1103/PhysRevLett.98.258101.
11. Barabás G, D'Andrea R, Stump SM. Chesson's coexistence theory. Ecological Monographs. 2018;doi:10.1002/ecm.1302.
12. Chesson P. Updates on mechanisms of maintenance of species diversity. Journal of Ecology. 2018;106:1773–1794. doi:10.1111/1365-2745.13035.
