# Supplementary material for: Generalizing clusters of similar species as a signature of coexistence under competition
Source: PLoS Comput Biol. 2019 Jan 22;15(1):e1006688. doi: 10.1371/journal.pcbi.1006688 (PMC6358094; doi:10.1371/journal.pcbi.1006688)
Supplement: S1 Box — (PDF) [file pcbi.1006688.s002.pdf]

Generalizing clusters of similar species as a signature of coexistence under competition

Rafael D'Andrea[1,2*], Maria Riolo[2], Annette M Ostling[2,3]

1 Ecology and Evolutionary Biology, University of Michigan, Ann Arbor, Michigan, USA

2 Plant Biology, University of Illinois, Urbana-Champaign, Illinois, USA

* rdandrea@illinois.edu

S1 Box: Detecting clusters with k-means and the gap statistic

Here we describe our metric based on coupling the k-means clustering algorithm [1] with the gap statistic method [2]. Conceptually, we assess the degree of clustering by measuring the total trait distance between species in the same cluster, summed over all clusters. Smaller values indicate tighter clustering. However, we cannot use this index directly unless we know the number of clusters to look for, as the index naturally declines with the number of clusters considered. To circumvent this, we compare this total distance to that found in null communities, and find the number of clusters that maximizes the difference from the null. This maximal difference is the statistic.

Stepwise, we proceed as follows. We start with a candidate number of clusters, k. Out of all possible ways to assign the observed species to k clusters, the k-means algorithm finds the one that minimizes within-cluster trait dispersion: $D_k = \sum_C \sum_{i,j \in C} n_i n_j d_{ij}$, where C refers to a cluster ($1 \leq C \leq k$), n_i is the abundance of species i, d_{ij} is the trait distance between species i and j. We define the clustering index $F_k = -\log(D_k)$, such that F_k is high for low dispersion D_k. We then repeat this step across a set of 5,000 null communities, and the difference in F_k between the observed community and the mean null value is the gap for k clusters,

$G_k = F_k - \overline{F}_{k,\mathrm{null}}$. We then test a range of numbers of clusters and find the one that maximizes the gap. This maximal value (the peak in the gap curve in Fig 1B) is the gap statistic, $G = \max(G_k)$, and the value of k at which it occurs is the estimated number of clusters K. Note that the gap step is needed because F_k will always increase with k: as we increase the number of clusters, the average cluster becomes smaller and therefore within-cluster dispersion decreases. By comparing against null communities, the gap method selects the number of clusters that most increases the clustering index beyond those expectations.

We then perform the same routine on each of the null communities to obtain a null distribution of gap statistics, from which we extract significance (p-value) and standardized effect size (z-score). The z-score is $Z = (G - \mu)/\sigma$, where $\mu = \frac{1}{5000}\sum_{\mathrm{nulls}} G_{\mathrm{null}}$ is the mean of the null gap statistics and $\sigma^2 = \frac{1}{5000}\sum_{\mathrm{nulls}}(G_{\mathrm{null}} - \mu)^2$ is the variance. The p-value is the proportion of null communities with a higher gap statistic than the observed community, $P = \frac{1}{5000}\sum_{\mathrm{nulls}} I(G_{\mathrm{null}} > G)$, where the indicator function I is 1 if its argument is true, and zero otherwise.

Fig 1 shows two test communities, and the respective results from our metric. The first community (Fig 1A) is constructed to contain four clusters, whereas in the second community (Fig 1C) abundances are unrelated to traits. The metric correctly diagnoses both (compare Fig 1B, D).

References

1. MacQueen JB. Some Methods for classification and Analysis of Multivariate Observations. 5th Berkeley Symposium on Mathematical Statistics and Probability 1967. 1967;1(233):281–297. Available from: `http://projecteuclid.org/euclid.bsmsp/1200512992`.

2. Tibshirani R, Walther G, Hastie T. Estimating the number of clusters in a data set via the gap statistic. Journal of the Royal Statistical Society: Series B (Statistical Methodology). 2001;63:411–423. Available from: `http://onlinelibrary.wiley.com/doi/10.1111/1467-9868.00293/abstract`.

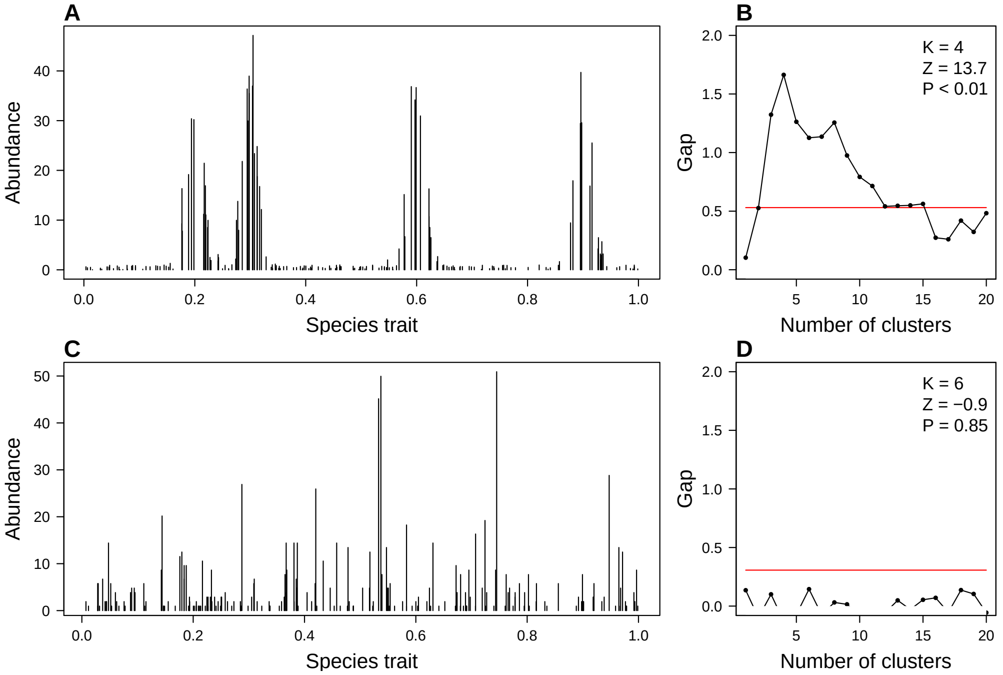

Fig 1. **A**: Hypothetical species assemblage representing a community under niche differentiation + immigration, with visible clustering. **B**: Plotting the dispersion gap against the number of clusters, we see a clear peak at $K = 4$ clusters. The gap value at the peak, $G_4 = 1.66$, is the gap statistic. It far exceeds the 95th percentile of the gap statistic across the null communities (red line), leading to a very high z-score and a very low p-value. **C**: Neutral community with same size as A, but abundances unrelated to traits. **D**: Corresponding gap curve indicates that no number of clusters gives a gap beyond null expectations. Witness the low z-score and high p-value. [*Numerical details*: Community A: 200 species traits were uniformly drawn between 0 and 1. We then assigned a species to each of 1,000 individuals, with probability of choosing species i given by $P_i = k \sum_{j=1}^{4} \exp(-|x_i - x_j^*|^2/2\sigma^2)$, where x_i is the trait of species i, $\mathbf{x}^* = \{0.2,\ 0.3,\ 0.6,\ 0.9\}$ are the centers of the four clusters, $\sigma = 0.02$ is the cluster width, and k is a normalizing constant ensuring $\sum_{i=1}^{200} P_i = 1$. Finally, abundances are boosted by a random small amount to represent immigration. Community C: we drew species abundances from a log-series distribution with parameters $N = 1,000$ and $\alpha = 50$, and assigned them to randomly chosen trait values.]
