# Supplementary material for: Generalizing clusters of similar species as a signature of coexistence under competition
Source: PLoS Comput Biol. 2019 Jan 22;15(1):e1006688. doi: 10.1371/journal.pcbi.1006688 (PMC6358094; doi:10.1371/journal.pcbi.1006688)
Supplement: S1 Appendix — (PDF) [file pcbi.1006688.s003.pdf]

Generalizing clusters of similar species as a signature of coexistence under competition

Rafael D'Andrea[1,2*], Maria Riolo[2], Annette M Ostling[2,3]

1 Ecology and Evolutionary Biology, University of Michigan, Ann Arbor, Michigan, USA

2 Plant Biology, University of Illinois, Urbana-Champaign, Illinois, USA

* rdandrea@illinois.edu

S1 Appendix: Mathematical formulation of the competition-colonization tradeoff model

This model was originally formulated as follows [1]

$$\frac{1}{p_i}\frac{\mathrm{d}p_i}{\mathrm{d}t} = f_i(1 - \sum_{j=1}^{i} p_j) - \sum_{j=1}^{i-1} f_j p_j - \mu, \tag{1}$$

where p_i is species i's relative abundance, and f_i is its fecundity (i.e. number of propagules per individual per unit time). The first term represents recruitment in available sites—i.e. all sites not currently occupied by species i or stronger competitors; the second term represents displacement by stronger competitors; the last term is intrinsic mortality μ, here assumed identical for all species.

We can gather the density-independent and the density-dependent terms separately:

$$\frac{1}{p_i}\frac{\mathrm{d}p_i}{\mathrm{d}t} = (f_i - \mu) - \sum_{j=1}^{S} \Theta_{ij}(f_i + f_j)p_j, \tag{2}$$

The step function Θ_{ij} (equal to 1 if $i > j$, 0 if $i < j$, and 0.5 if $i = j$) encodes the strict competitive hierarchy. It has been shown [2] that the strict hierarchy is unrealistic (because arbitrarily similar species will have large differences in competitive ability) and drastically inflates coexistence. Here we use instead a probabilistic, gradual hierarchy:

the higher the difference in rank, the higher the likelihood of displacement by the better competitor. We do this by replacing Θ_{ij} with the continuous function $\Gamma_{ij} = 0.5\,(1 - \tanh[s(f_j - f_i)])$, which is equal to 0.5 when $f_i = f_j$ and asymptotes to 1 and 0 when $f_i \gg f_j$ and $f_i \ll f_j$, respectively. The parameter s controls the steepness of the hierarchy, and hence the degree of coexistence. (We recover the step function when $s \to \infty$.)

Notice that Equation (2) is in the Lotka-Volterra shape, $\frac{1}{N_i}\frac{\mathrm{d}N_i}{\mathrm{d}t} = r_i - \sum_j A_{ij} N_j$, where the effective intrinsic growth rate $r_i = b_i - d_i$ is the difference between intrinsic fecundity and mortality. We use that correspondence in our stochastic formulation, where we place the density dependence in the deaths.

References

1. Kinzig aP, Levin S, Dushoff J, Pacala S. Limiting Similarity, Species Packing, and System Stability for Hierarchical Competition-Colonization Models. The American Naturalist. 1999 apr;153(4):371–383. Available from: http://www.jstor.org/stable/10.1086/303182.

2. D'Andrea R, Barabás G, Ostling AM. Revising the tolerance-fecundity trade-off; or, on the consequences of discontinuous resource use for limiting similarity, species diversity, and trait dispersion. The American Naturalist. 2013;181(4):E91–101. Available from: http://www.ncbi.nlm.nih.gov/pubmed/23535625.
