# Supplementary material for: Generalizing clusters of similar species as a signature of coexistence under competition
Source: PLoS Comput Biol. 2019 Jan 22;15(1):e1006688. doi: 10.1371/journal.pcbi.1006688 (PMC6358094; doi:10.1371/journal.pcbi.1006688)

Generalizing clusters of similar species as a signature of coexistence under competition

Rafael D'Andrea[1,2*], Maria Riolo[2], Annette M Ostling[2,3]

1 Ecology and Evolutionary Biology, University of Michigan, Ann Arbor, Michigan, USA

2 Plant Biology, University of Illinois, Urbana-Champaign, Illinois, USA

* rdandrea@illinois.edu

S2 Appendix: Detailed description of clustering metrics

Our method proceeds in the following general steps:

1. Given the data and a certain parameter value φ within a provided range $[\varphi_{\min}, \varphi_{\max}]$, calculate a clustering measure $F(\varphi)$.

2. Repeat step 1 in each of n null communities, $\widetilde{F}_i(\varphi)$, $i = 1, 2, \ldots, n$, and take the mean, $\overline{\widetilde{F}}(\varphi) = \frac{1}{n}\sum_i \widetilde{F}_i(\varphi)$.

3. The difference $G(\varphi) = F(\varphi) - \overline{\widetilde{F}}(\varphi)$ is the "gap" at that parameter value.

4. Repeat steps 1-3 for all parameter values within $[\varphi_{\min}, \varphi_{\max}]$.

5. The gap statistic is the extremum (maximum or minimum) of the gap function, $G = \operatorname*{extr}_{\varphi} G(\varphi)$.

6. Obtain a z-score and a p-value by repeating steps 1-5 on each of the null communities, which provides a null distribution for G. The z-score is then $Z = (G - \mu_{\widetilde{G}}) \,/\, \sigma_{\widetilde{G}}$ and the p-value is $P = \frac{1}{n}\sum_i I(\widetilde{G}_i \geq G)$, where $\widetilde{G}_i$ is the gap statistic obtained for the i-th null assemblage, $\mu_{\widetilde{G}}$ and $\sigma_{\widetilde{G}}$ are the mean and standard deviation of those values, and index function I is 1 if its argument is true and 0 otherwise.

Note that this recipe works with different clustering measures, F. Here we use two: k-means dispersion [1] and Ripley's K function [2, 3]. In the k-means version, the parameter φ is the number of clusters, and the value $\hat{\varphi}$ at which the gap is maximal is the estimated number of clusters in the community. In the Ripley's K version, φ is the trait distance between pairs of individuals, and the value at which the gap is minimal is the average distance between clusters.

Note also that we can use any number of traits to describe our species, as long as we can define a "distance" between species (e.g. Euclidean distance in a high-dimensional trait space, or simple trait differences on a single trait axis).

Gap statistic via k-means

Here the parameter φ is the candidate number of clusters k, and we use the k-means clustering algorithm [1]. For a given k, the algorithm finds the partition of individuals into k groups that minimizes the within-group dispersion. Let D_k be the total pairwise squared distance between members of each group, i.e., $D_k = \sum_{C=1}^{k} \sum_{i,j \in C} n_i n_j d_{ij}^2$, where C refers to a cluster, n_i is the abundance of species i, d_{ij} is the trait distance between species i and j. The k-means algorithm finds the species-to-cluster assignment that minimizes D_k. (In our model, all conspecific individuals have the same trait value, and therefore necessarily belong in the same cluster. For efficiency we modified the k-means algorithm to arrange all individuals of a species together.)

The algorithm starts with randomly chosen trait values in the local community as possible cluster centers, then puts species into the cluster whose center is the closest to them, then recalculates cluster centers, and so on until the algorithm converges or subsequent changes in D_k fall below a specified threshold. Since the result can depend on the starting point, we carry out this procedure from a variety of randomly chosen cluster centers, and take the final cluster arrangement with the lowest D_k across different starting points. If a cluster ended up empty in this approach, the arrangement was not included in calculating the minimum D_k, and was replaced with a different one. We assessed the number of starting points needed by verifying that the resulting D_k changed little if more starting points were added. This number was relatively similar across model scenarios. We used a single starting point for 1 cluster (for which the

arrangement is independent of the number of starting points), 1,000 starting points for 2-5 clusters, 5,000 starting points for 6-10 clusters, 10,000 starting points for 11-15 clusters, and 100,000 starting points for 16-20 clusters. The exception was the habitat partitioning model, where empty clusters occurred frequently, and hence we used fewer starting points to maintain reasonable computational time.

We then set $F(\varphi) = F_k = \log(1/D_k)$ as the clustering measure to be maximized with the Gap statistic. Note that it does not make sense to compare F_k directly across different k's because F_k necessarily increases with k, as the average within-cluster distance is always lower for higher numbers of clusters. By comparing against null assemblages, the gap method finds the biggest increase in goodness of fit *beyond* what is expected from the increase in k. The reason we use $\log(1/D_k)$ rather than simply $1/D_k$ is that the expected increments in $1/D_k$ with increasing k are multiplicative rather than additive (see [4] for more details).

See S1 Box for a step-by-step recipe for this metric.

Gap statistic via Ripley's K

The approach with Ripley's K [2] is to count the number of pairs of individuals within each distance in trait space and determine whether or not it deviates significantly from what we would expect if the same set of abundances were randomly distributed among the present species. In particular, we look for significant open spaces between clusters, i.e. distances at which Ripley's K is surprisingly low. The parameter φ here is therefore the trait distance d.

Ripley's K function at distance d is defined as $K(d) = \frac{\sum_{i \neq j} I(d_{ij} < d) N_i N_j}{\sum_{i \neq j} N_i N_j}$, where N_i is the abundance of species i, d_{ij} is the distance in trait space between species i and j, and function $I(.)$ is the indicator function, equal to 1 if its argument is true and 0 otherwise. We then calculate Ripley's K in each of our null assemblages, and define the Ripley's K clustering measure as the standardized K function: $F(d) = K(d)/\sigma_{\widetilde{K}(d)}$, where $\sigma_{\widetilde{K}(d)}$ is the standard deviation of the null values. The standardization compensates for the natural increase in the variance of $K(d)$ at large d (see Fig 1). The gap function is then $G(d) = K(d)/\sigma_{\widetilde{K}(d)} - \mu_{\widetilde{K}(d)}/\sigma_{\widetilde{K}(d)}$.

Because we are interested in significantly low counts, we define our Ripley gap

statistic as the minimum value of $G(d)$ across candidate distances, $G = \min_{d} G(d)$. This is analogous to our implementation with the k-means algorithm (see Table 1 for a direct comparison between the two implementations).

Fig 1 shows the metric at work on an example of a Lotka-Volterra community, a community under neutral competition and environmental filtering, and a purely neutral community. When the community has significant open spaces (Fig 1A), i.e. a lower G than the null communities, it suggests multiple clusters in trait space of high abundance species, with a dearth of species in between them. This is what we would expect from a community shaped by niche differentiation. When instead it is significantly lacking in gaps (Fig 1B), that indicates a single clump of abundant species, as could be expected when the environment filters for a single trait value.

Note that while k-means and Ripley's K can characterize a community in terms of clustering structure, the gap statistic tells us whether that structure is significant compared to a null model. Furthermore, while clustering measures will typically depend on a parameter (number of clusters in the case of k-means, distance between clusters in the case of Ripley's K), the gap statistic removes the parameter by comparing the data to the null model across the parameter's range.

References

1. MacQueen JB. Some Methods for classification and Analysis of Multivariate Observations. 5th Berkeley Symposium on Mathematical Statistics and Probability 1967. 1967;1(233):281–297. Available from: `http://projecteuclid.org/euclid.bsmsp/1200512992`.

2. Ripley BD. The Second-Order Analysis of Stationary Point Processes. Journal of Applied Probability. 1976;13(2):255–266.

3. Dixon PM. Ripley's K function. In: El-shaarawi AH, Piegorsch WW, editors. Encyclopedia of Environmetrics. vol. 3. Chichester: John Wiley & Sons; 2002. p. 1796–1803.

4. Tibshirani R, Walther G, Hastie T. Estimating the number of clusters in a data set via the gap statistic. Journal of the Royal Statistical Society: Series B

(Statistical Methodology). 2001;63:411–423. Available from: http://onlinelibrary.wiley.com/doi/10.1111/1467-9868.00293/abstract.

Table 1. Comparison of our two implementations of the gap method. Both apply the gap method using different measures of dispersion. $W(k)$ is the k-means dispersion for k clusters; $K(d)$ is Ripley's K at trait distance d; $F(k)$ $(F(d))$ is the dispersion for k clusters (distance d); I is the indicator function, equal to 1 if its argument is true and zero otherwise; N_i is the abundance of species i; $G(k)$ $(G(d))$ is the gap function for k clusters (distance d); $\mu_{\widetilde{X}}$ and $\sigma_{\widetilde{X}}$ are the mean and standard deviation of quantity X taken across the null communities; $\hat{d}$ is the distance at which $G(d) = G$. n is the number of null communities.

	**k-means**	**Ripley's K**
Clustering measure	$F_k = \log(1/W(k))$	$F(d) = K(d)/\widetilde{\sigma}_K(d)$
Gap function	$G_k = F_k - \mu_{\widetilde{F}_k}$	$G(d) = F(d) - \mu_{\widetilde{F}(d)}$
Gap statistic	$G = \max_k G_k$	$G = \min_d G(d)$
Estimated number of clusters	k such that $G_k = G$	Number of abundance-peaks within distance $\hat{d}$ of each other
Z-score	$Z = (G - \mu_{\widetilde{G}}) \,/\, \sigma_{\widetilde{G}}$	$Z = (G - \mu_{\widetilde{G}}) \,/\, \sigma_{\widetilde{G}}$
P-value	$P = \frac{1}{n} \sum_i I(\widetilde{G}_i \geq G)$	$P = \frac{1}{n} \sum_i I(\widetilde{G}_i \geq G)$

Fig 1. **Top**: Abundances plotted against trait values in a sample replicate of (**A**) the Lotka-Volterra niche model, (**B**) environmental filtering without a niche mechanism, and (**C**) the neutral model. **A1-C1**: Ripley's K vs. trait distance in the same replicates. Values for the observed community are plotted in black. The %95 confidence interval of $K(d)$ among the null communities (obtained by reshuffling abundances across species) is shaded in gray. **A2-C2**: same results with mean null value at each distance subtracted out, $K(d) - \widetilde{\mu}_K(d)$. **A3-C3**: rescaled by the variance at each distance $G(d) = K(d)/\widetilde{\sigma}_K(d) - \widetilde{\mu}_K(d)/\widetilde{\sigma}_K(d)$. The red line is the threshold for a significantly low gap statistic ($p < 0.05$), and the triangle indicates the distance with the most surprisingly low density of pairs, i.e. the distance d where the Ripley gap statistic G is achieved, $G(d) = G$.

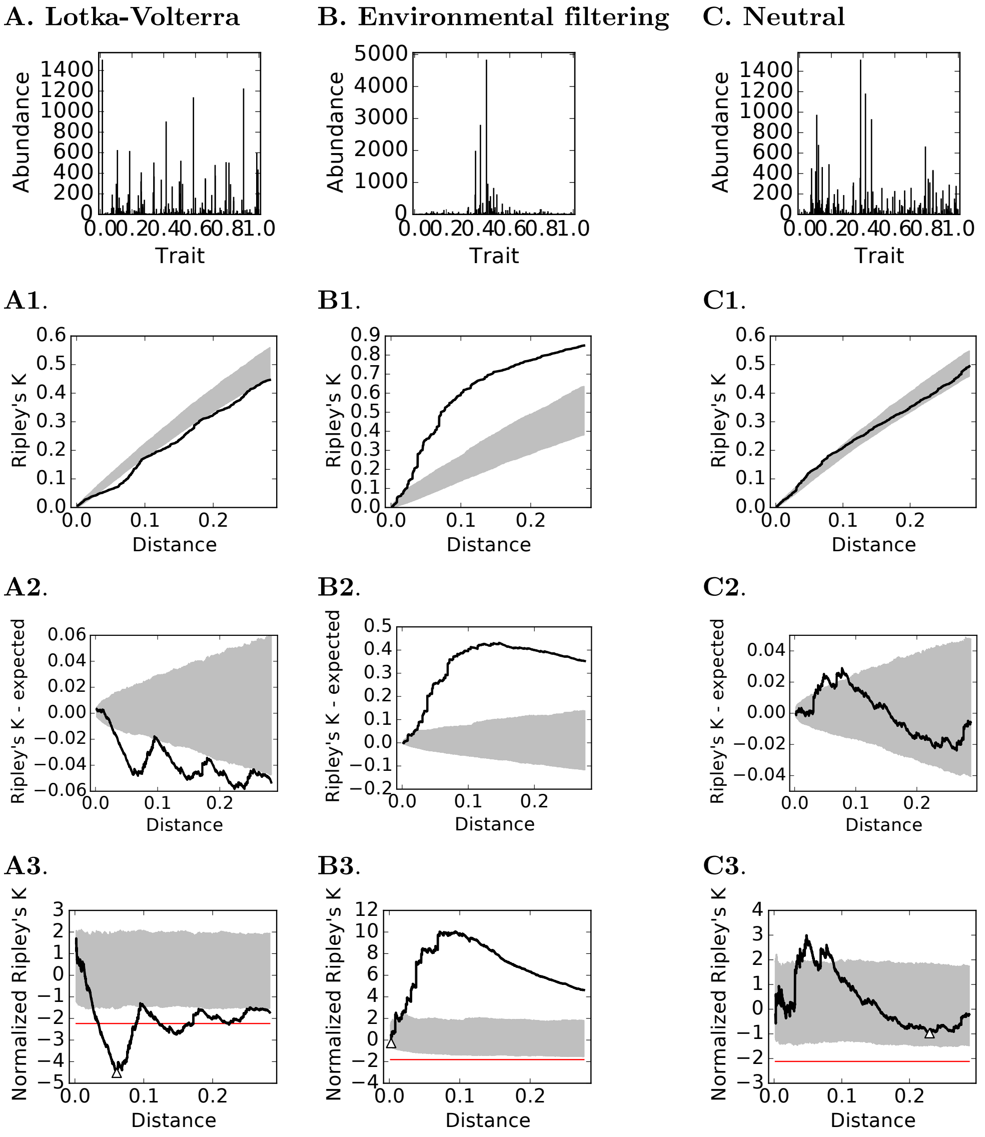

Supplement: S2 Appendix — (PDF) [file pcbi.1006688.s004.pdf]
