# Supplementary figures and images for: Generalizing clusters of similar species as a signature of coexistence under competition
Source: PLoS Comput Biol. 2019 Jan 22;15(1):e1006688. doi: 10.1371/journal.pcbi.1006688 (PMC6358094; doi:10.1371/journal.pcbi.1006688)

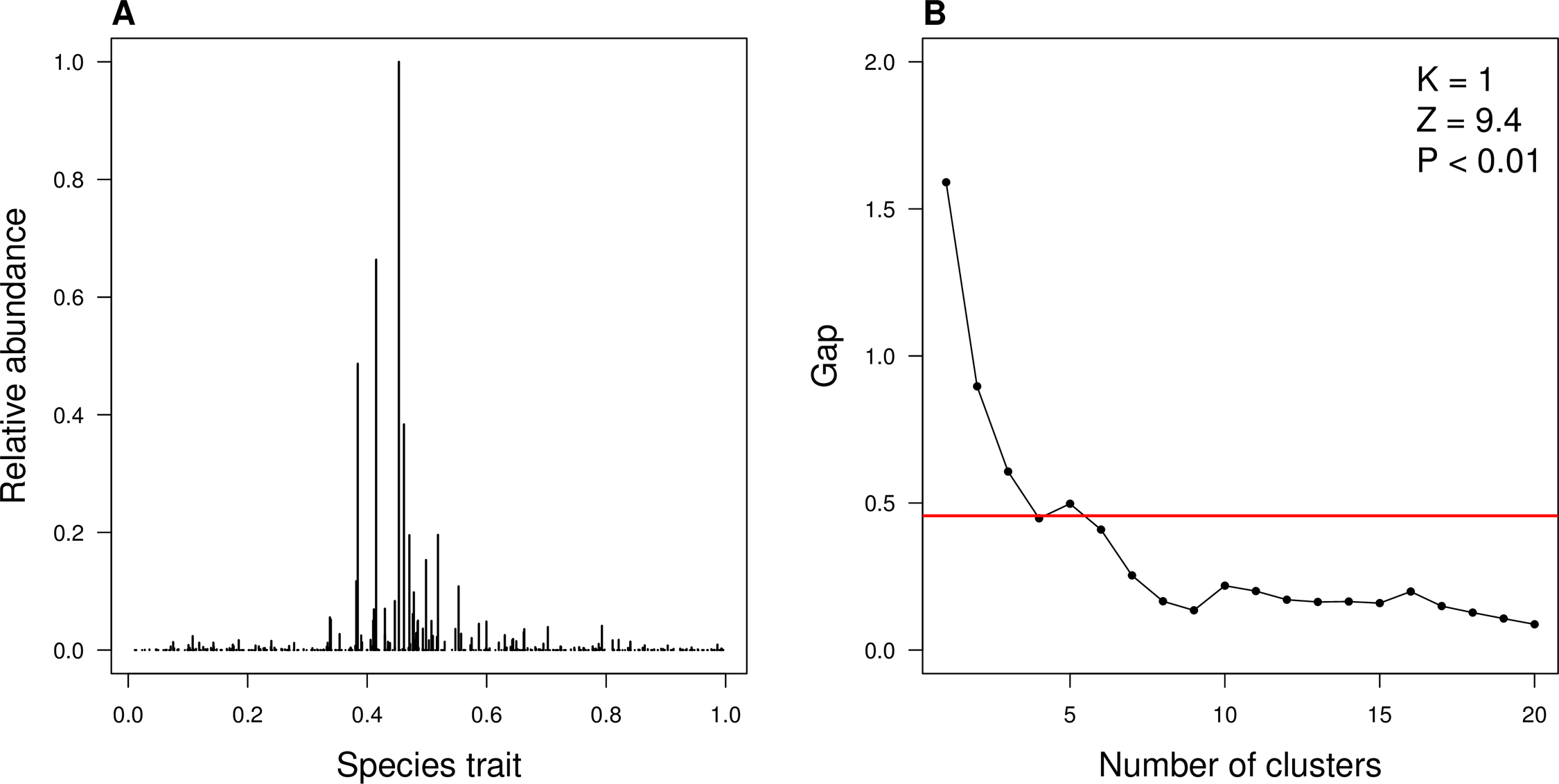

Supplement: S1 Fig — A: Example simulation outcome of Lotka-Volterra stochastic dynamics with neutral competition coefficients, αij = 1, such that there is no niche mechanism, and intrinsic growth rates given by ri = xi(1 − xi), where xi is the trait of species i. The latter represents environmental filtering for species with intermediate traits. B: Corresponding gap curve, showing gap index for each number of clusters between 1 and 20, has a clear maximum at 1 cluster. The estimated number of clusters is therefore K = 1. The gap statistic is well above the 95% quantile of the null distribution (red line), indicating significance at P < 0.05. All 100 replicates of this scenario were clustered, with a single cluster detected by the k-means metric in all cases. (TIF) [file pcbi.1006688.s005.tif]

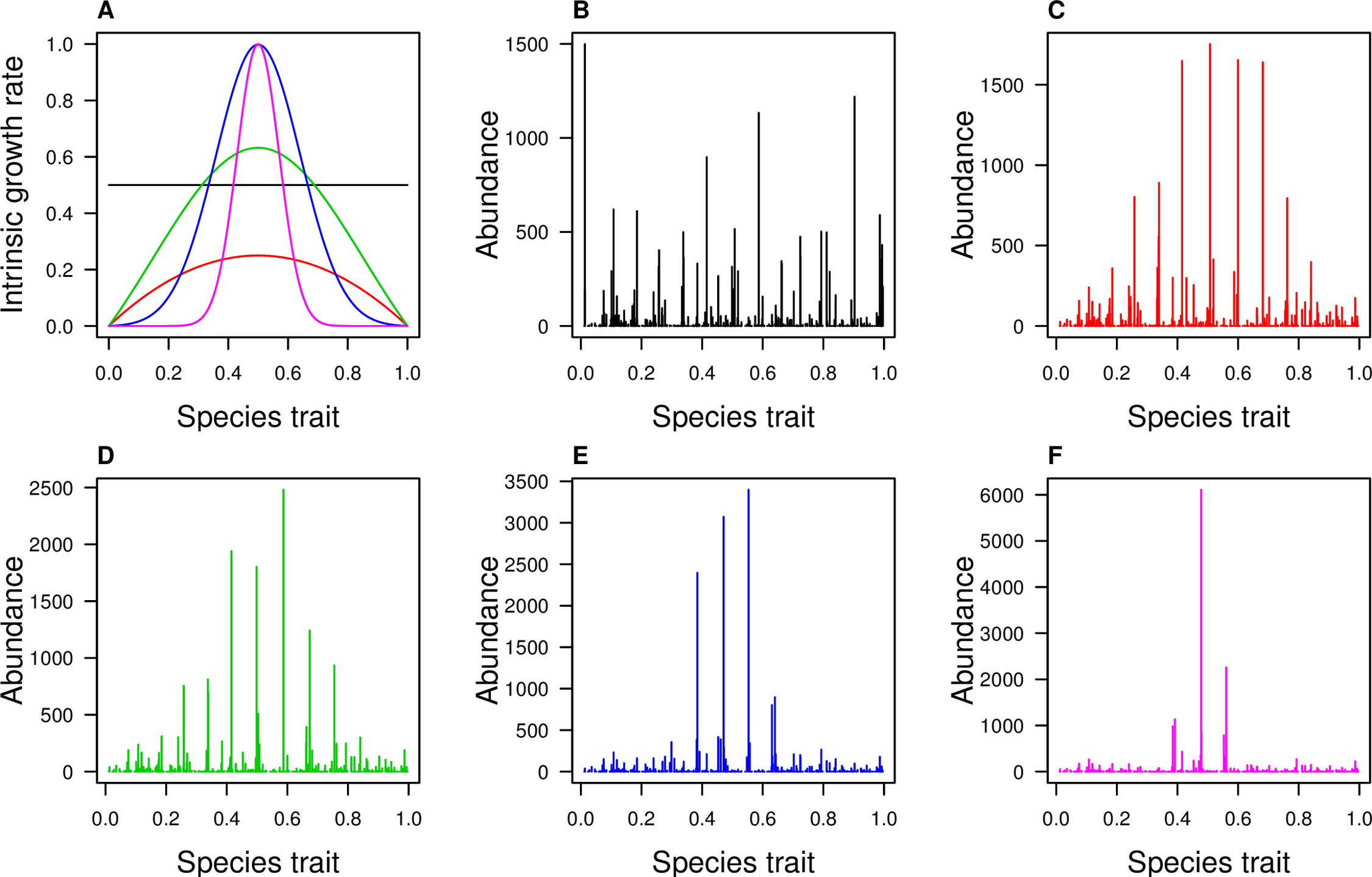

Supplement: S2 Fig — A: We implemented environmental filters via a modal relationship between intrinsic growth rate r and species traits xi as follows: r = 0.5 (no filtering, black); ri = xi(1 − xi) (red); ri = exp(−(xi − 0.5)2/σ2) with σ = 0.5 (green), 0.2 (blue), 0.1 (magenta). B: Under no filtering, the community shows no overarching abundance trend. C-F: Under increasing filter intensity, communities show increasingly steeper abundance trends. Our metrics detected multiple clusters in 10/10 replicates of B-D, but only 2/10 in E and 1/10 in F, with the remaining replicates having a single cluster. When no niche mechanism is at play, such that species compete neutrally but still differ by intrinsic growth rates, all replicates result in a single cluster centered on the species with the highest intrinsic growth rate. (TIF) [file pcbi.1006688.s006.tif]

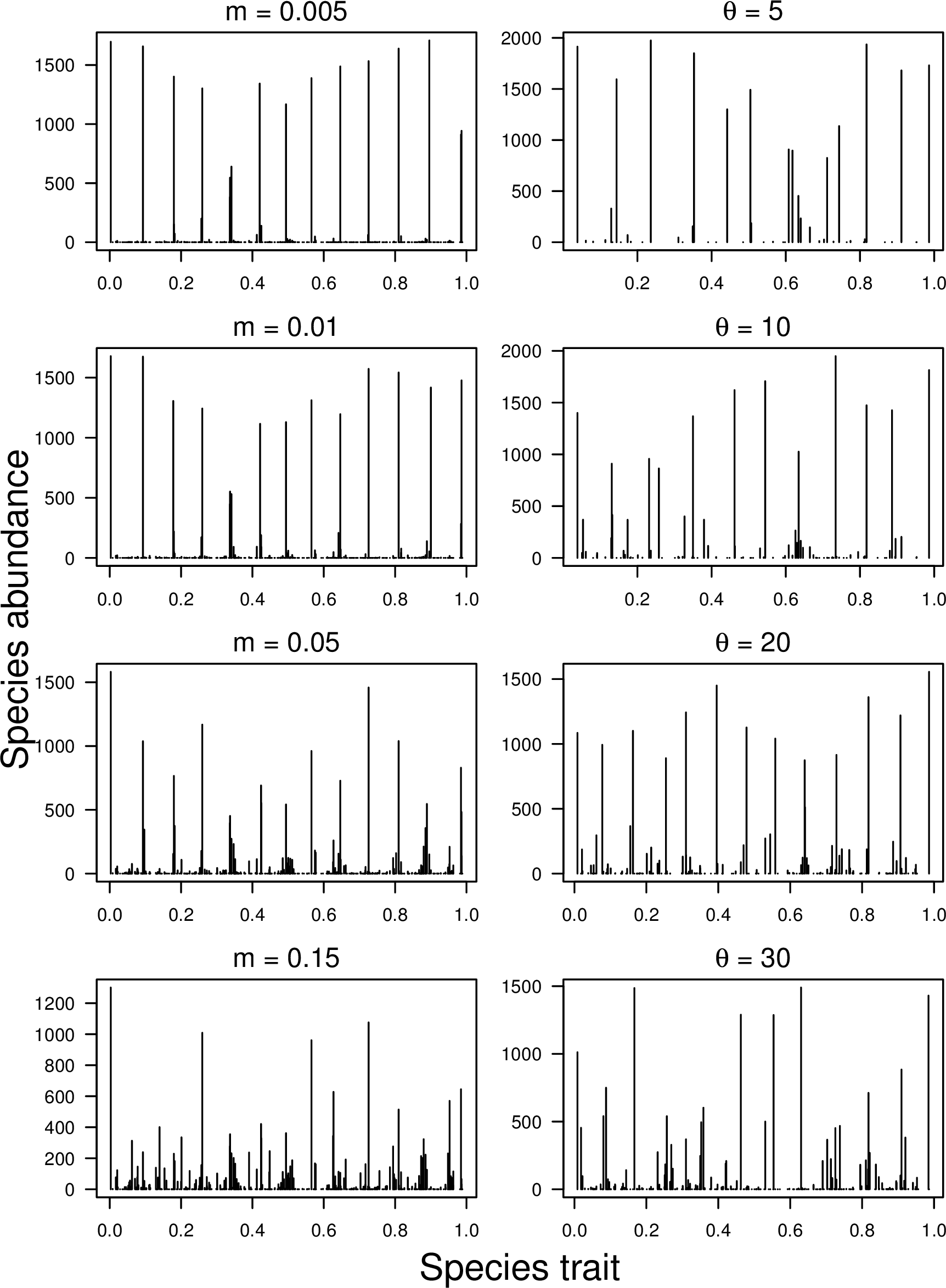

Supplement: S3 Fig — Example Lokta-Volterra communities with increasing immigration rate m (left) and regional diversity parameter θ (right). (TIF) [file pcbi.1006688.s007.tif]

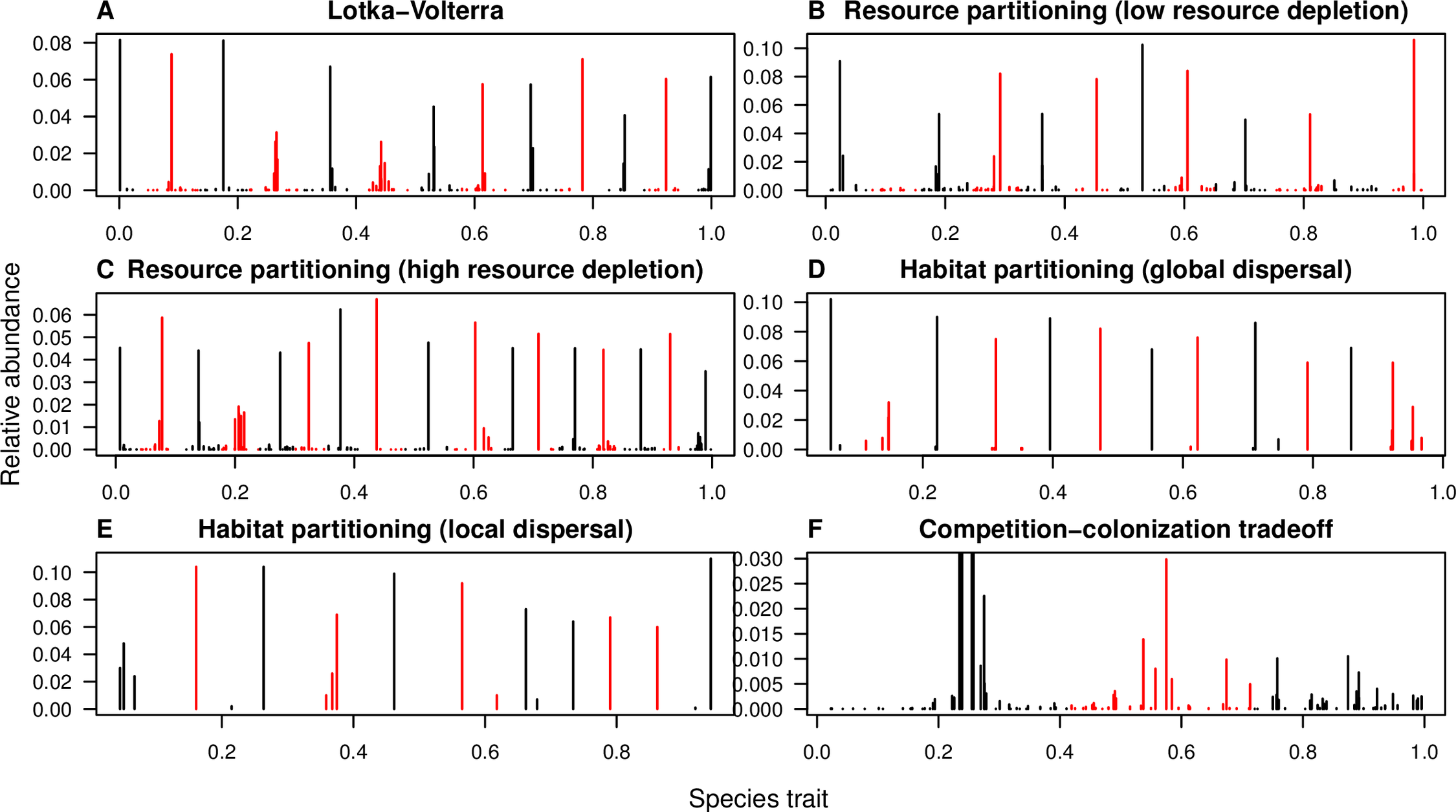

Supplement: S4 Fig — Comparing panels A-F against their respective m = 0.08 counterparts in Fig 2 (panels A, C-G with matching titles), we see that communities show a similar number of clusters but fewer species per cluster, and gaps between clusters are more rarified. (TIF) [file pcbi.1006688.s008.tif]

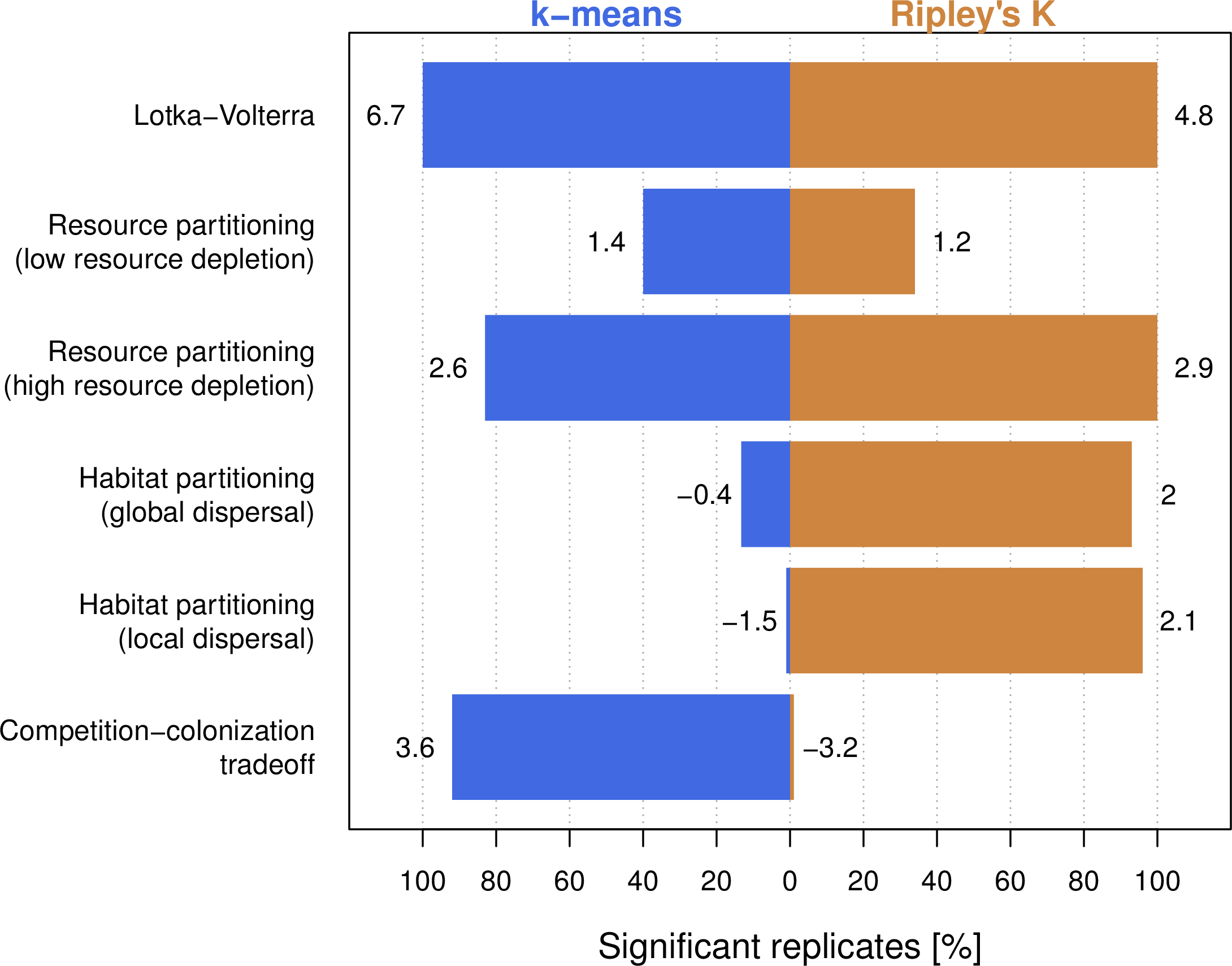

Supplement: S5 Fig — Results are similar to m = 0.08 (compare with Fig 3), although z-scores and significance are often higher, particularly for resource-partitioning communities, and habitat-partitioning communities under global dispersal. This indicates that higher immigration is drowning the pattern in these niche scenarios. (TIF) [file pcbi.1006688.s009.tif]

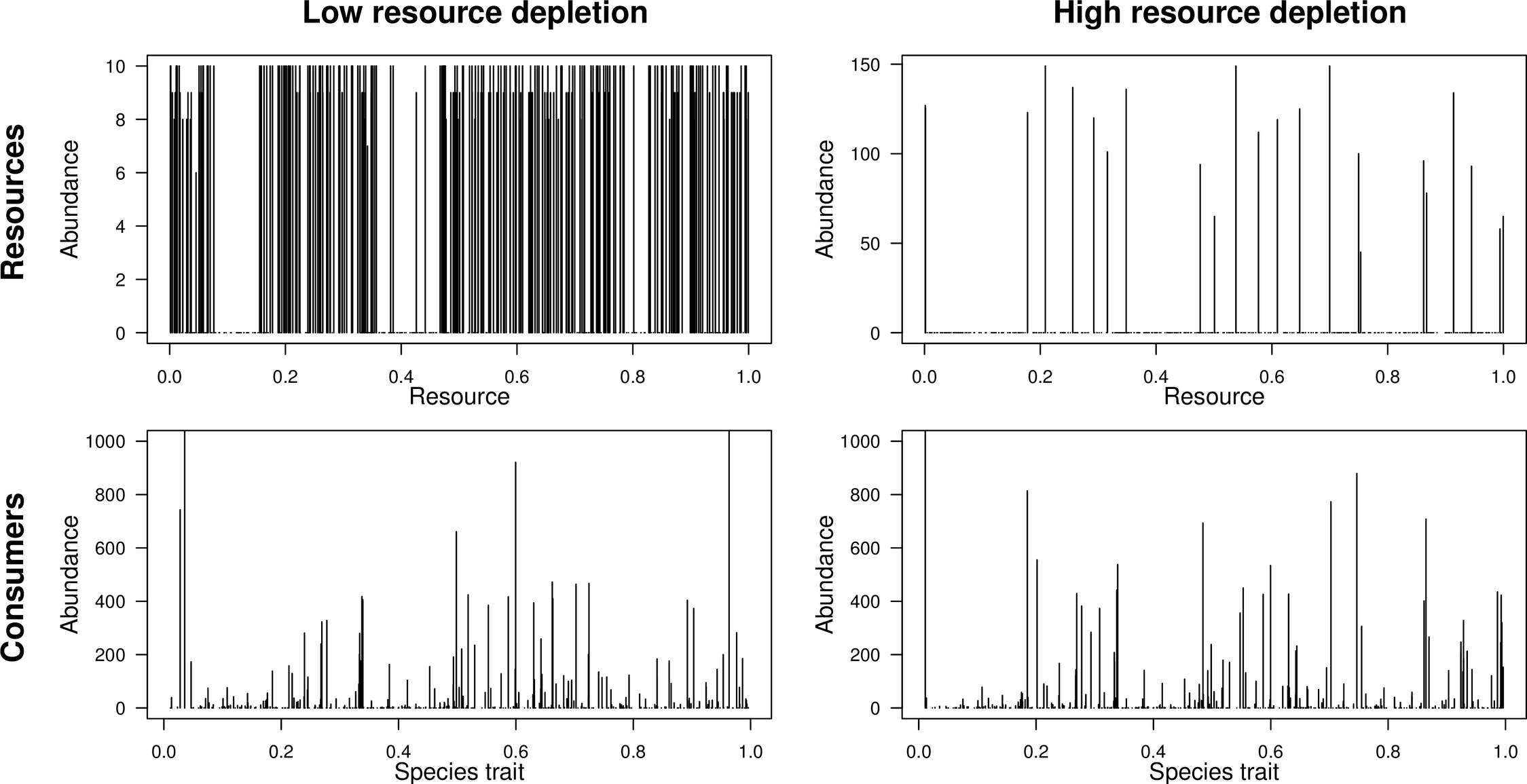

Supplement: S6 Fig — Example communities where consumers partition resources, under low and high resource depletion (left and right columns, respectively). Resources are shown on top, consumers at the bottom. Under high resource depletion, gaps left by resource extirpation cause corresponding gaps among consumer species. These gaps strengthen the clustering pattern relative to the low depletion scenario. (m = 0.08, c. 400 regional species). (TIF) [file pcbi.1006688.s010.tif]

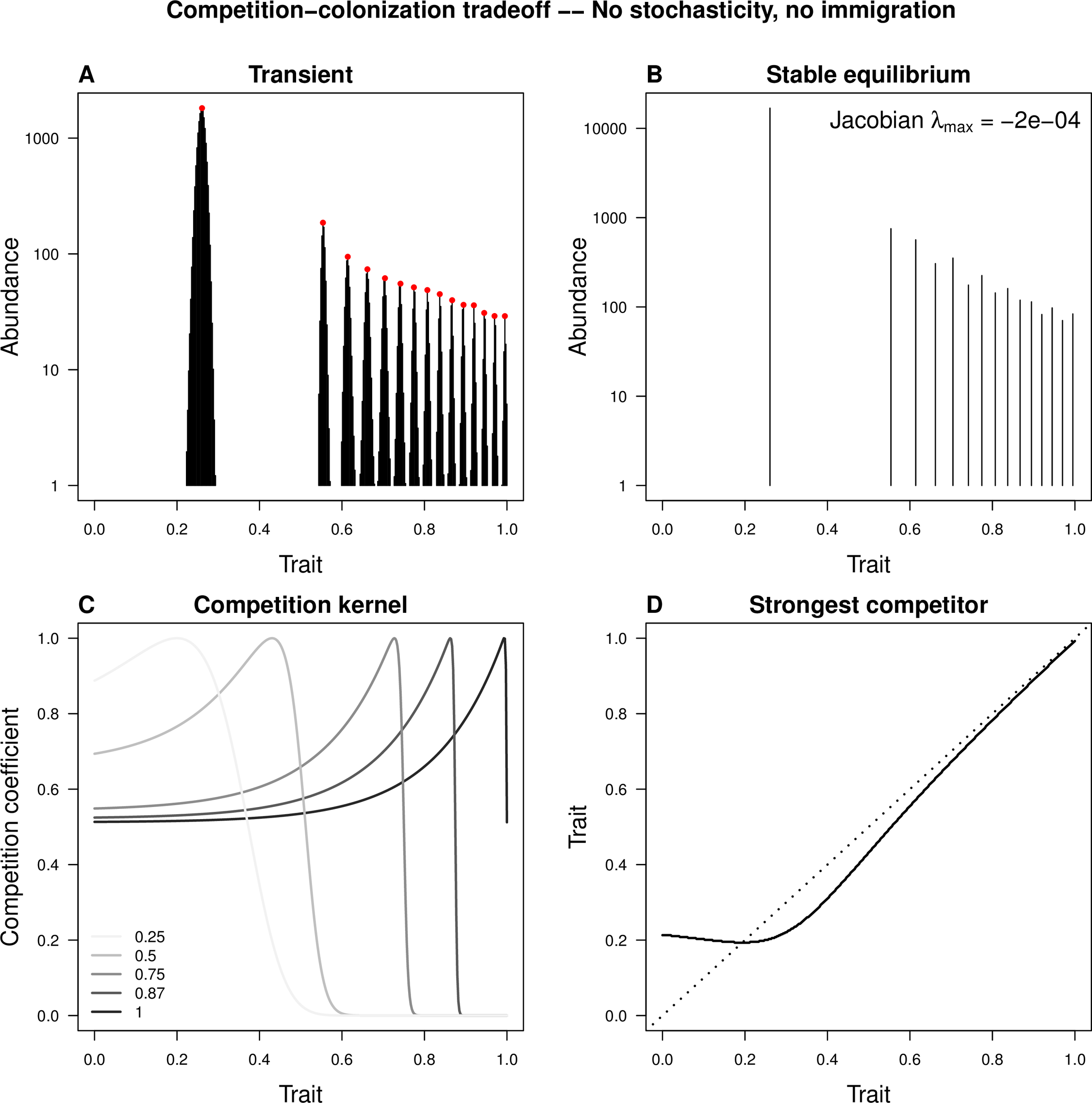

Supplement: S7 Fig — A. Transient state shows visible clustering. B. Equilibrium state, showing abundances of the coexisting species, which are those that dominate their respective clusters in the transient state (marked with red dots in A). Legend shows maximum eigenvalue of the Jacobian of the equilibrium, indicating dynamical stability of the equilibrium. C. Competition kernel shows the strongest competitors on focal species, whose traits are shown in legend, are species with similar traits, despite the competitive hierarchy. This contributes to stabilization of the community. D. For each species (traits plotted on x-axis), the position of the peak of the competition kernel (i.e. the species with the strongest net competitive impact on it) is plotted on the y-axis. The proximity of the curve to the one-to-one line (dotted line) throughout the trait axis shows that the competition-colonization tradeoff stabilizes the community, thus acting as a niche mechanism. The kernel maxima plotted here also explain the absence of coexisting species below trait ≃ 0.2, as the strongest competitors of those species have higher fecundity than themselves (solid curve is above dotted line), thus being both more competitive and more fecund. The wide gap between the first cluster and the other clusters is also reflected in the relatively larger distance from the one-to-one curve in that region of the trait axis. (TIF) [file pcbi.1006688.s011.tif]
